# The Hyperbolically Symmetric Black Hole

**DOI:** 10.3390/e27080831

**Published:** 2025-08-05

**Authors:** Luis Herrera, Louis Witten

**Affiliations:** 1 Instituto Universitario de Física Fundamental y Matemáticas, Universidad de Salamanca, 37007 Salamanca, Spain; 2 Independent Researcher, Ringhouse-180 E 1. Jefferson Road, Rockville, MD 20847, USA; lwittenw@gmail.com

**Keywords:** black holes, exact solutions, general relativity

## Abstract

We describe some properties of the hyperbolically symmetric black hole (hereafter referred to as the HSBH) proposed a few years ago. We start by explaining the main motivation behind such an idea, and we determine the main differences between this scenario and the classical black hole (hereafter referred to as the CBH) scenario. Particularly important are the facts that, in the HSBH scenario, (i) test particles in the region inside the horizon experience a repulsive force that prevents them from reaching the center, (ii) test particles may cross the horizon outward only along the symmetry axis, and (iii) the spacetime within the horizon is static but not spherically symmetric. Next, we examine the differences between the two models of black holes in light of the Landauer principle and the Hawking results on the eventual evaporation of the black hole and the paradox resulting thereof. Finally, we explore what observational signature could be invoked to confirm or dismiss the model.

## 1. *HSBH* Versus *CBH*

Before contrasting the HSBH with the CBH, we first present the general arguments leading us to propose the HSBH model [1].

### 1.1. Why the HSBH Model?

As is well known in the CBH scenario, the spacetime outside the horizon is described by the Schwarzschild solution [2], whose line element in polar coordinates reads for R>2M (in relativistic units and with signature +2) as follows:

(1) $$\begin{array}{ccc} ds^{2} & = & -\left(1-\frac{2M}{R}\right)dt^{2}+\frac{dR^{2}}{\left(1-\frac{2M}{R}\right)}+R^{2}d\Omega^{2},\\ d\Omega^{2} & = & d\theta^{2}+\sin^{2}\theta d\phi^{2}, \end{array}$$

where *M*, which measures the total mass-energy of the source, is the only parameter of the solution.

Such a metric being static and spherically symmetric admits the four Killing vectors

(2) $$\begin{array}{c} X_{(0)}=\partial_{t},\;X_{\left(2\right)}=-\cos\phi \partial_{\theta }+\cot\theta \sin\phi \partial_{\phi },\\ X_{\left(1\right)}=\partial_{\phi },\;X_{\left(3\right)}=\sin\phi \partial_{\theta }+\cot\theta \cos\phi \partial_{\phi }. \end{array}$$

Also, as is well known, the singularity appearing in (1) for R=2M is not a real physical singularity, and the solution may be analytically extended to the whole spacetime (including the region inside the horizon), as shown in [3,4,5,6].

However, the fact is that the spacetime within the horizon is necessarily non-static. Maintaining the static form of the Schwarzschild metric (in the whole spacetime) is incompatible with the possibility of removing the coordinate singularity appearing on the horizon in the line element [7]. This is related to the fact that static observers cannot be defined inside the horizon (see [8,9] for a discussion on this point). A simple way to see why this comes about consists of finding out the form of the null cone at any point inside the horizon by solving the equation

(3) ds=0.

The solution to the above equation shows that all the null rays generating the null cone (inside the horizon) converge to the center of symmetry, implying that anything within the null cone (including the massless particles along the null cone border) should reach the center (see [8,9,10] for a discussion on this point).

Although most of our colleagues are satisfied with this picture, we are not. Our concern is generated by the intuitive idea that any dynamical process should relax to an equilibrium state after some finite proper time. In such a case, we should be able to provide a global static description of the spacetime.

To the best of our knowledge, the first to call attention to this issue was Rosen [7], who proposed excluding the region R<2M because, in Rosen’s words, “…the surface R=2M represents the boundary of physical space and should be regarded as an impenetrable barrier for particles and light rays” (see page 233 in [7]).

Sharing Rosen’s concern about the physical properties of the region R<2M, we proposed in [1] an alternative way to overcome the above-mentioned situation.

Thus, we proposed describing the region R>2M via the Schwarzschild solution (1); however, instead of excluding the region R<2M, we assume that the spacetime in that region is defined by the line element (with signature −2)

(4) $$\begin{array}{ccc} ds^{2} & = & \left(\frac{2M}{R}-1\right)dt^{2}-\frac{dR^{2}}{\left(\frac{2M}{R}-1\right)}-R^{2}d\Omega^{2},\\ d\Omega^{2} & = & d\theta^{2}+\sinh^{2}\theta d\phi^{2}. \end{array}$$

The above metric is static; therefore, it admits the time-like Killing vector $X_{\left(0\right)}$, and, furthermore, it admits the three Killing vectors

(5) $$\begin{array}{c} Y_{\left(2\right)}=-\cos\phi \partial_{\theta }+\coth\theta \sin\phi \partial_{\phi },\\ Y_{\left(1\right)}=\partial_{\phi },\;Y_{\left(3\right)}=\sin\phi \partial_{\theta }+\coth\theta \cos\phi \partial_{\phi }, \end{array}$$

implying that it is not spherically symmetric but hyperbolically symmetric.

The above three Killing vectors (5) define the hyperbolical symmetry. For applications of this kind of symmetry, see [11,12,13,14,15,16,17,18,19,20,21,22,23,24,25,26,27,28,29,30,31,32,33] and the references therein.

The situation may be summarized as follows: We have already seen that, when extending the Schwarzschild metric to the region inner to the horizon, which means keeping the spherical symmetry, one should abandon staticity; this is the picture for the CBH. However, we wish to keep staticity within the horizon. Accordingly, we propose abandoning sphericity, which leads to the HSBH picture.

A short comment is in order at this point: In [1], we arrive at (4) from (1) through the transformation θ→iθ. In doing so, the signature of the resulting line element (4) is different from the signature of (1). However, obviously, (4) is a static solution to the vacuum Einstein equations independently on its signature. In other words, the transformation mentioned above is just a technicality, and we may assume the region inside the horizon to be described by a hyperbolically symmetric metric with the same signature as the Schwarzschild line element describing the region outside the horizon. Thus, we do have a change in symmetry across the horizon but not necessarily a change in its signature.

### 1.2. Geodesics in HSBH

In [19], a general study of geodesics in the spacetime described by (4) is presented (see also [25]), leading to some interesting conclusions that highlight great differences between the behavior of a test particle in the HSBH and the results emerging from the CBH, particularly the following:

- Inside the region R<2M, the gravitational force appears to be repulsive.
- As a consequence of the above, test particles never reach the center.
- Unlike the CBH, test particles can cross the horizon outward, but only along the θ=0 axis.

Based on the last point above, it could be (rightly) argued that the object described by the HSBH is not really “black” since matter may cross the horizon outwardly. However, in order to keep the link between the two scenarios (the HSBH and CBH), we decide to keep the term “black”.

The results above are supported by the expression of the four-acceleration ($a^{\mu }$) of a static observer in the frame of (4). Indeed, $a^{\mu }$ represents the inertial radial acceleration that should be applied to the frame in order to keep the frame static by opposing the gravitational acceleration exerted on it.

Next, for a static observer, the four-velocity $U^{\mu }$ is proportional to the Killing time-like vector [8]. Then, for (4), we obtain

(6) $$U^{\mu }=\left[\frac{1}{{\left(\frac{2M}{r}-1\right)}^{1/2}},0,0,0\right],$$

producing for the four-acceleration $a^{\mu }\equiv U^{\beta }U_{;\beta }^{\mu }$ within the region inner to the horizon

(7) $$a^{\mu }=\left[0,-\frac{M}{r^{2}},0,0\right],$$

whereas for the region outside the horizon, defined by (1), the four-velocity vector of a static observer reads

(8) $$U^{\mu }=\left[\frac{1}{{\left(1-\frac{2M}{r}\right)}^{1/2}},0,0,0\right],$$

producing for the four-acceleration

(9) $$a^{\mu }=\left[0,\frac{M}{r^{2}},0,0\right].$$

From a simple inspection of (9), we see that the gravitational force in the region outer to the horizon is attractive, as it follows from the positive value of the acceleration (directed radially) in this region, while it is repulsive in the region inner to the horizon, as it follows from (7). The repulsive nature of gravitation in the spacetime described by (4) is characteristic of hyperbolical spacetimes and is at the origin of the peculiarities of the orbits within the horizon in the HSBH.

### 1.3. Flow of Information and Landauer Principle

The Landauer principle [34] asserts that, in the process of erasing one bit of information, some energy must be dissipated, a lower bound of which is given by

(10) △E=kTln2,

where *k* is the Boltzmann constant, and *T* denotes the temperature of the environment (see also [35,36]).

Its relevance stems from the fact that it leads in a natural way to an “informational” reformulation of thermodynamics, which, in turn, allows for establishing a link between information theory and different branches of science [37,38,39,40].

The link between the Landauer principle and general relativity is discussed in detail in [41]. Here, we resort to some results found in that reference to carry on our discussion on the differences between the HSBH and CBH.

Thus, a mass given by

(11) $$M_{bit}=\frac{kT}{c^{2}}\ln2,$$

was assigned to any bit of information, where *c* is the speed of light [42].

This issue (the mass of information) has been discussed by several authors (see [43,44,45] and the references therein).

Our goal in this subsection consists of contrasting the behavior of the information flow across the horizon in the gravitational field of a static mass in the HSBH and CBH scenarios. To this end, first, we need an expression for the Landauer principle in the presence of a gravitational field.

The presence of a gravitational field in the weak field approximation produces a change in the total amount of minimal dissipated energy, which is given by [46]

(12) $$△E=\frac{kT\left(1+\frac{\phi }{c^{2}}\right)}{c^{2}}\ln2,$$

where ϕ denotes the Newtonian (negative) gravitational potential. Thus, in the presence of a gravitational field, the Landauer principle is modified by replacing the temperature *T* by the Tolman temperature [47], which, in the weak field limit, reads $T\left(1+\frac{\phi }{c^{2}}\right)$.

Let us recall that, in presence of a gravitational field, thermodynamic equilibrium is ensured by the condition that the Tolman temperature is constant. This result, which is a consequence of the inertia of thermal energy, emerges naturally in the relativistic transport equations proposed in [48,49,50], and it should hold in any physically admissible, relativistic transport equation.

The generalization of (10) for a gravitational field of an arbitrary intensity reads

(13) $$\Delta E=kT\sqrt{|g_{tt}|}\ln2,$$

whereas the expression for the mass of a bit becomes

(14) $$M_{bit}=\frac{kT\sqrt{|g_{tt}|}}{c^{2}}\ln2,$$

where $g_{tt}$ is the tt component of the metric tensor, and the fact is used that the Tolman temperature is defined by $T\sqrt{|g_{tt}|}$, which, in the weak field limit, becomes $T(1+\frac{\phi }{c^{2}})$.

Let us now consider the CBH scenario.

In this case, if we admit that a bit of information is endowed with a mass according to (14), then, since nothing can cross the horizon outwardly, it follows that no information can cross the horizon outwardly. This conclusion agrees with the results obtained by Hawking about the radiation of a CBH due to quantum effects [51] and its eventual evaporation, as well as with the fact that such radiation is completely thermal (i.e., it conveys no information) [52]), which leads to a contradiction known as the information loss paradox.

Indeed, the Hawking result implies that a pure quantum state evolves into a mixed state (the thermal radiation), which, of course, contradicts the principle of unitary evolution. Since the Hawking radiation is thermal, it appears that all information about the collapsing object is lost forever.

In spite of the intense research work devoted to this issue in recent decades, no satisfactory resolution of this quandary has yet been proposed.

Let us next consider the HSBH scenario.

As mentioned before, in such a case, the crossing of massive particles through the horizon outwardly is allowed along the θ=0 axis, thereby implying the existence of a flux of information from the inside of the horizon to the outside. Thus, in this scenario, no information loss paradox appears, since even if the Hawking radiation is thermal, information may leave the collapsed object.

Furthermore, in this latter picture, the energy flow along the axis θ=0 associated with the change in information within the horizon could be, in principle, observable.

Thus, invoking the Landauer principle in the study of the global picture of the Schwarzschild black hole may lead to observable consequences, which could help to elucidate the quandary about the real nature of the Schwarzschild black hole.

## 2. Observational Evidences

Finally, we would like to mention some observational projects that could be determinant to dismiss or confirm the HSBH scenario.

The most important of these endeavors seems to be a study based on the information provided by the Event Horizon Telescope (EHT) Collaboration [53,54,55], which provides observations of shadow images of the gravitationally collapsed objects at the center of the elliptical galaxy M87 and at the center of the Milky Way Galaxy. Such observations are expected to provide important data on strong fields [56,57,58], which could be used to obtain constraints for the parameters of the solutions describing the geometry surrounding compact objects, e.g., black hole spacetimes in modified and alternative theories of gravity [59,60,61,62,63], naked singularities, and classical GR black holes with hair or immersed in matter fields [64,65,66,67].

It should be noted that, although the region exterior to the horizon is described by the usual Schwarzschild metric, we might expect some imprints on the shadows from the material ejected along the symmetry axis in the HSBH.

Another possible source of information to support the HSBH picture could be the observation and modeling of extragalactic relativistic jets.

These are highly energetic phenomena that have been observed in many systems (see [68,69,70,71] and the references therein), usually associated with the presence of a compact object. One of their characteristic properties is a high degree of collimation (besides the extremely high energies involved).

So far, no consensus has been reached concerning the basic mechanism explaining these two features of jets (collimation and high energies); therefore, it is legitimate to speculate that the HSBH could be considered a possible engine behind the jets.

Indeed, let us recall that, in the HSBH scenario, test particles may cross the horizon outward, but only along the θ=0 axis, thereby explaining the collimation. However, as it follows from (7), the strength of the repulsive gravitational force acting on the particle as r→0 increases as $\frac{1}{r^{2}}$, thereby explaining the high energies of the particles bouncing back from regions close to r=0.

Obviously, at this level of generality, this is just speculation. A solid theory for relativistic jets would require a much more detailed setup based on astronomical observations.

## 3. Discussion and Conclusions

Above, we exposed the main features of the HSBH and contrasted them with those of the CBH. We started by explaining that our main motivation for proposing the HSBH was the need to describe the region interior to the horizon by a static spacetime, which, in turn, forced us to abandon the assumption of spherical symmetry in that region. The analysis of the geodesic structure within the horizon exhibited profound differences from the corresponding structure in the CBH.

As we showed, such differences are related to the fact that the nature of gravity within the horizon happens to be repulsive, as a consequence of which test particles never reach the center. This feature is perhaps the most relevant difference between the HSBH and CBH.

The origin of the repulsive nature of gravitation within the horizon should be found in the presence of negative mass (energy). This is indeed the case, as shown in the studies of fluid distributions endowed with hyperbolical symmetry (see [18,21,22,30] for details).

It is worth mentioning that the existence of negative mass (energy) in gravitational physics has a long and venerable history, at both the classical [10,72,73,74,75,76,77] and quantum levels [78,79,80,81,82,83,84].

We next invoked the Landauer principle to illustrate the differences between the HSBH and CBH concerning the possible flow of information and the discussion of the Hawking results in both scenarios. It follows in a natural way that no information loss paradox appears in the HSBH.

Finally, we call attention to a new line of investigations involving observations of shadow images of the gravitationally collapsed outcome, aiming to identify the nature of different compact objects. This could provide arguments to support (or dismiss) the HSBH picture. Also, the idea has been put forward about the possible use of the HSBH as the engine of extragalactic jets.

Before concluding, we would like to highlight three important issues that we believe deserve further investigation:

- The two manifolds describing the inner and outer parts of the horizon do not match smoothly in the Darmois sense [85]. This implies that there is a shell on the horizon, whose physical and mathematical properties are described by the Israel conditions [86]. It would be interesting to delve deeper into this issue and find out how these properties affect the HSBH scenario.
- It would be interesting to find out whether a “thermodynamic” approach *a la Bekenstein* may also be applied to the HSBH.
- The HSBH scenario implies a change in symmetry across the horizon; is there any physical explanation for this (e.g., a phase transition)?

## Data Availability

Data are contained within the article.
